# La Crosse Virus Infection of Human Keratinocytes Leads to Interferon-Dependent Apoptosis of Bystander Non-Infected Cells In Vitro

**DOI:** 10.3390/v12030253

**Published:** 2020-02-25

**Authors:** Maria A. Cruz, Griffith D. Parks

**Affiliations:** Burnett School of Biomedical Sciences, College of Medicine, University of Central Florida, Orlando, FL 32827, USA; mariaangelica.cruzzamora@ucf.edu

**Keywords:** La Crosse virus, keratinocytes, interferon, bystander cell death

## Abstract

Resident cells in the skin serve as the first innate line of defense against insect-borne pathogens, but the role of these cell types in promoting or limiting arbovirus replication is not completely understood. Here, we have examined the outcome of infection of cultured human keratinocyte cells with La Crosse virus (LACV), using a spontaneously transformed cell line, HaCaT. In single cycle infections, keratinocyte HaCaT cells supported rapid and high level LACV replication, resulting in high virus yields and extensive caspase-dependent cell death. By contrast, multi-cycle LACV replication in HaCaT cells was restricted by an antiviral response elicited by the production of both IFN-β and IFN-λ. During low multiplicity LACV infections, HaCaT cell death was seen in non-infected bystander cells. Media from LACV-infected cells induced caspase-dependent killing of naïve non-infected HaCaT cells, and this bystander cell death was relieved by IFN-β neutralizing antibodies or by an inhibitor of JAK-STAT signaling. Naïve HaCaT cells showed dose-dependent killing by treatment with exogenous IFN-β but not IFN-λ. Our data suggest a model whereby keratinocytes produce IFNs which limit virus spread through both antiviral signaling and by induction of bystander cell death of potential new target cells for infection.

## 1. Introduction

The skin serves as the first line of defense against infection by insect-borne pathogens such as arboviruses, both as a physical barrier and as a source of antiviral responses which have the potential to limit dissemination [1]. As such, the capacity of skin resident cells to support arbovirus replication or, alternatively, to limit virus spread could be an important determinant of pathogenesis. Here, we have examined the host cell and antiviral interactions of La Crosse virus (LACV), a prototype negative strand RNA arbovirus with human keratinocytes in vitro.

The Peribunyavirus LACV is primarily transmitted to humans by the *Aedes triseratus* mosquito. Typically, LACV infection results in a mild febrile illness, however, in a small subset of pediatric cases, LACV infection leads to meningoencephalitis, seizures, and paralysis [2,3]. La Crosse virus is the leading cause of pediatric arboviral encephalitis in the United States [4,5,6]. The actual number of LACV infection cases is estimated to be much higher than reported, since infection numbers are hard to calculate due to underreporting of non-neurological cases that lack distinct symptoms [7,8]. Currently, there are no approved therapeutics or vaccines for LACV infections. Due to the increased range of mosquitos and the introduction of new potential vectors to endemic areas, LACV is considered an emerging threat in the Eastern United States [6,9,10].

The replication and pathogenesis of LACV has been extensively studied in mouse model systems, which show the same age dependence as humans for infection and subsequent neurological disease—young mice are susceptible to LACV infection whereas adult mice are resistant [11]. When LACV is introduced subcutaneously into mice, there is initial virus replication at the site of delivery and dissemination into blood where a plasma viremia can be observed. From the blood, the virus enters the brain through unknown routes, where it replicates primarily in neurons, leading to cell death and neurological symptoms [12,13,14]. Type I interferon (IFN) pathways can play a role in protecting mice from lethal bunyavirus infections [11], acting in a potential range of cell types to limit dissemination or regulate neuroinvasion [15]. In mice, myeloid dendritic cells (DC) are a key source of IFN induction by LACV that can control neurological disease, being primarily driven by endosomal Toll-like receptors (TLRs) and retinoic acid-inducible gene I (RIG-I) detection of viral RNA [11]. Other key components in the IFN response in non-myeloid cells include signaling through mitochondrial antiviral-signaling protein (MAVS) to activate interferon regulatory factor (IRF)-3, IRF-5 and IRF-7 [16]. Type I IFN signaling can then induce expression of antiviral IFN-stimulated gene (ISG) products, including protein kinase R (PKR), IFN-induced protein 44 (IFI44), and viperin, which have been shown to inhibit replication of some bunyaviruses [17]. In the case of LACV, the GTP binding protein MxA has been shown to prevent the accumulation of viral transcripts and proteins, possibly through trapping of viral nucleoprotein in perinuclear vesicles [18,19,20]. 

Since arboviruses are inoculated directly into the dermis and epidermis by mosquitos, there has been strong interest in how dermal cell types, such as keratinocytes and fibroblasts, can play roles in the outcome of these infections. For example, it has been shown that keratinocytes are the primary site of replication for West Nile virus [21]. By contrast, Chikungunya virus (CHIKV) replication appears to be restricted in keratinocytes, but this virus replicates to high levels in dermal fibroblasts [22,23,24,25]. In Dengue virus (DV) infections, initial replication can occur in the dermal layer, where subsequent inflammatory responses driven by local immune cells (e.g., DC) as well as keratinocytes can enhance recruitment of blood immune cells which can then potentially serve as viral reservoirs for dissemination in the host [26,27,28,29]. Among dermal cell types, keratinocytes are of particular interest in the early stages of some viral infections, since: (1) they express basal or inducible levels of many pattern recognition receptors such as RIG-I and Toll-like receptors that can recognize a wide variety of pathogens [30,31,32], and (2) they can express a range of immunomodulatory cytokines including interleukin (IL)-1, IL-6, IL-8, tumor necrosis factor family proteins (TNFs), and IFNs in response to pathogen exposure [33,34,35,36,37,38,39].

Given the importance of dermal-resident cells as an initial site for arbovirus infection, we have examined the outcome of LACV infection of human keratinocytes cells in culture. Here, we show that keratinocytes are both, highly permissive to LACV infection and support rapid virus growth and extensive cell death. However, during multi-cycle LACV infections of keratinocytes, IFN responses can limit spread through the population of cells. Unexpectedly, we show that IFN-β induced by LACV infection also contributes to the killing of bystander non-infected neighboring cells. 

## 2. Materials and Methods 

### 2.1. Cells, Viruses, and Infections

The HaCaT keratinocyte cell line was obtained from AddexBio Technologies Inc. (San Diego, CA, USA). Vero cells were provided by Robert Lamb (Northwestern University, Evanston, IL, USA). Cultures of HaCaT and Vero cells were grown in Dulbecco modified Eagle medium (DMEM) supplemented with 10% heat inactivated bovine calf serum (HI FBS, Hyclone, Logan, UT). La Crosse virus (LACV) was kindly provided by Andrew Pekosz (Johns Hopkins Bloomberg School of Public Health, Baltimore, MD) and was grown in C6/36 cells (ATCC^®^) in Leibovitz’s L-15 Medium (without HI FBS) supplemented with 10% tryptose broth and bovine serum albumin (BSA). 

Viral titers were determined by plaque assay on Vero cells. Briefly, 6-well plates of Vero cells at 90% confluency were infected with serial dilutions of LACV in DMEM supplemented with 10% BSA. After 1 h incubation, cells were washed with PBS and overlayed with a 1:1 mixture of 0.6% agarose and DMEM supplemented with 2% FBS. After six days, overlay was removed and cells were fixed and stained with a 0.37% formalin and 0.1% crystal violet solution.

HaCaT cells were used at passages below 10 to maintain consistency in culture. Infections were performed at a multiplicity of infection (MOI) of 5, 0.5 or 0.05 plaque forming units (PFU)/cell by incubating virus and cells in DMEM containing 2% HI FBS. As a control, cells were also mock- infected by incubating with media only. After 1 h of incubation, cells were washed, and media was replaced with DMEM 10% HI FBS. Hours post-infection (hpi) were counted from the time the virus was initially added to the cells. 

### 2.2. Western Blotting

HaCaT cells were plated at 4 × 10^4^ cells/well in a 24-well plate and were infected with LACV at a MOI of 5. Cells were lysed in 100 µL of 1% sodium dodecyl sulfate (SDS) at the indicated timepoints. Samples were analyzed by sodium dodecyl sulfate-polyacrylamide gel electrophoresis (SDS-PAGE), followed by Western blotting with rabbit polyclonal sera against the N protein of LACV at 1:5000 dilution. Polyclonal anti-N serum was produced using N protein expressed in bacteria from PCR fragments generated from the LACV genome. The full protocol is available upon request through the corresponding author. Blots were visualized by horseradish peroxidase-conjugated antibodies and chemiluminescence (Thermo Fisher Scientific, Waltham, MA, USA).

### 2.3. Cell Viability and Caspase Assays

Cells cultured in 24-well plates or 48-well plates were treated as indicated in each figure legend. Media and trypsinized adherent cells were centrifuged and analyzed for annexin V binding (BD Bioscience, San Jose, CA, USA) and propidium iodide (BD Bioscience) staining as described by the manufacturer. Cells were analyzed by flow cytometry using the CytoFLEX (Beckman Coulter, Brea, CA, USA) and 10,000 independent events were analyzed using CytExpert software (Beckman Coulter). 

Alternatively, cytotoxicity assays were performed in 96-well white plates (Corning, Corning, NY, USA) using CytoTox-Glo reagent (Promega, Madison, WI, USA) according to the manufacturer’s instructions. Data are expressed as a fold change over mock-infected cells analyzed in parallel. Functional caspase assays were performed in 96-well white plates (Corning) using Caspase-Glo 9, 8, or 3/7 assays (Promega) according to the manufacturer’s instructions. Data are expressed as a fold change over mock-infected cells analyzed in parallel. 

### 2.4. ZVAD Treatment

Cells were plated at 3.5 × 10^4^ cells/well in a 24-well plate and treated with Z-VAD-FMK (Promega) or DMSO as a vehicle control diluted in DMEM supplemented with 10% HI FBS at a concentration of 40 μM for 30 min. Cells were then infected as described above. Virus was removed and replaced with Z-VAD-FMK at 20 μM diluted in DMEM supplemented with 10% HI FBS.

### 2.5. Supernatant Preparation

At 80% confluency, 6-well plates of cells were infected with LACV at a MOI of 5 PFU/cell. At times indicated in the figure legends, media was collected and treated with UV light from a germicidal G30T8 bulb for 15 min to inactivate LACV. To confirm virus inactivation, naïve HaCaT cells were treated with UV-supernatant. After 24 h, cells were trypsinized, washed, fixed, and permeabilized (eBioscience, San Diego, CA, USA) according to manufacturer’s protocol. Cells were stained with an anti-LACV Gc antibody 807.31ab (kindly provided by Andrew Pekosz) and Alexa Fluor^®^ 488, a fluorophore conjugated secondary antibody (Invitrogen, Carlsbad, CA, USA). Cells were analyzed by flow cytometry using the CytoFLEX system (Beckman Coulter), and 10,000 independent events were recorded and analyzed by using CytExpert software. 

### 2.6. Reverse Transcription and Real-Time PCR

HaCaT cells cultured in 6-well dishes were infected at a MOI of 5 PFU/cell. At timepoints indicated in the figure legends, cells were collected in TRIzol^®^ followed by RNA extraction (Invitrogen). To produce cDNA, 1 μg of total RNA was used with TaqMan^®^ Reverse Transcription Reagents (Applied Biosystems, Foster City, CA, USA) as described in the manufacturer’s instructions. Quantitative real-time PCR was performed using Bio-Rad CFX Connect Real-Time (Bio-Rad, Hercules, CA, USA) and Fast SYBR^®^ FAST Green Master Mix (Applied Biosystems, Foster City, CA, USA). Relative gene expression was determined using CFX Manager 3.1 Software (Bio-Rad) and the following primers (Table 1):

### 2.7. Interferon, Ruxolitinib and Neutralizing Antibody Treatment

HaCaT cells plated at 3 × 10^4^ cells/well were treated with varying concentrations of IFN-Lambda (IL-29 or IFN-λ1) or IFN Beta (IFN-β) (PBL Assay Sciences, Piscataway, NJ, USA) for 16 h. All dilutions were carried out in DMEM supplemented with 10% HI FBS.

HaCaT cells were plated at 4 × 10^4^ cells/well in a 24-well plate and treated with DMSO as vehicle control or Ruxolitinib (Invivogen, San Diego, CA, USA) at a concentration of 1 μM for 16 h. Cells were infected for 1 h before washing and replacement with DMEM supplemented with 10% HI FBS and 1 μM Ruxolitinib or DMSO vehicle control. Alternatively, cells were infected and then treated with neutralizing antibodies against IL29 (Invivogen), IL28a or IFN-λ2 (Invivogen), and Human IFN-λ1 receptor (IFNLR; PBL Assay Science), or IFN-β (Millipore, Burlington, MA, USA) at the concentrations stated in the figure legends. Corresponding isotype antibodies were used as negative controls.

### 2.8. Cell Viability and Virus Infection Quantification

HaCaT cells were cultured in a 24-well plate. Media and trypsinized adherent cells were centrifuged and stained with Zombie Red™ (BioLegend, San Diego, CA, USA) to quantify viability. Cells were then fixed, permeabilized (eBioscience) and stained with anti-LACV Gc antibody 807.31ab (kindly provided by Andrew Pekosz) and Alexa Fluor^®^ 488 (Invitrogen). Cells were analyzed by flow cytometry using the CytoFLEX system (Beckman Coulter), and 10,000 independent events were recorded and analyzed by using CytExpert software.

### 2.9. Statistical Analyses

Values are the mean of three replicates and experiments were performed at least twice. Statistical analysis was performed using GraphPad Prism 8, student’s *t*-test or a two-way ANOVA. In all figures, * indicates *p*-value < 0.05, ** indicates *p*-value < 0.01, and *** indicates *p*-value < 0.001.

## 3. Results

### 3.1. HaCaT Cells Are Permissive to LACV Infection

**Figure 1:**
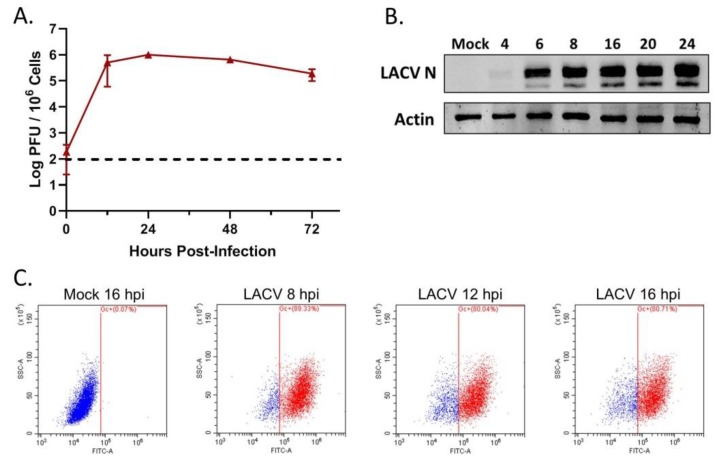
LACV can productively infect human keratinocyte cells. HaCaT cells were infected or mock- infected with LACV at a MOI of 5 PFU/cell. (**A**) At the indicated hpi, media was collected and viral titers were determined by plaque assay. Dotted horizontal line indicates limit of detection. (**B**) At the indicated timepoints, cells were lysed and analyzed by Western blotting for β-actin or for LACV N or (**C**) cells were analyzed by flow cytometry for LACV Gc expression. Dot plots shown are representative of three replicates.

To determine if LACV can productively infect human keratinocyte cells, the HaCaT cell line was infected with LACV at a MOI of 5 PFU/cell. At 12, 24, 48 and 72 h post-infection (hpi), cell supernatant was collected and viral titers were quantified by plaque assay. As shown in Figure 1A, viral titers increased rapidly over time with a peak titer of ~10^6^ PFU/mL at 24 hpi. HaCaT cells were infected at a high MOI and viral protein expression was measured by Western blot analysis of LACV nucleocapsid protein (N) accumulation. As shown in Figure 1B, N protein was detected in lysates as early as 6 hpi. As an alternative assay, HaCaT cells were mock- infected or LACV-infected at a MOI of 5 PFU/cell, and cells were collected at 8, 12, and 16 hpi for analysis of total viral Gc expression by flow cytometry using an antibody against the LACV Gc protein. Consistent with LACV N expression, almost 90% of LACV-infected cells expressed the Gc glycoprotein by 8 hpi with no substantial increase at later timepoints (Figure 1C). Together, these data indicate that HaCaT cells are permissive to high multiplicity LACV infection and can produce high levels of progeny virions.

### 3.2. LACV Infection Induces Caspase-Dependent Cell Death in HaCaT cells

**Figure 2:**
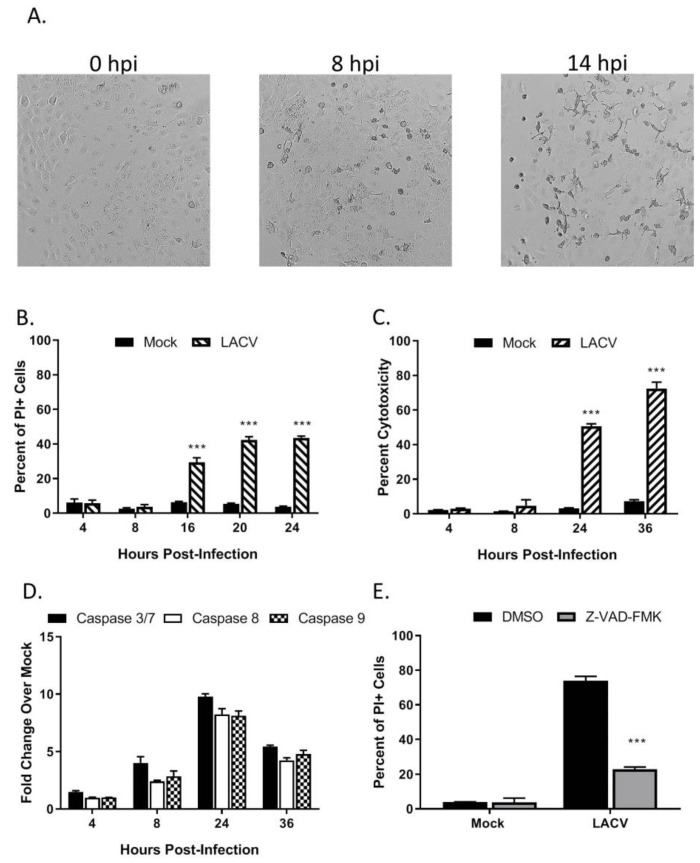
LACV infection of human keratinocytes induces caspase-dependent cell death, (**A**–**D**) HaCaT cells were infected at a MOI 5 PFU/cell. (**A**) At the indicated hpi, cells were imaged at 10× magnification, and representative bright field images are shown. Alternatively, cell viability and cytotoxity was determined by (**B**) PI staining or (**C**) Cytotox-Glo assay. (**D**) Caspase activity in cell lysates was determined by Caspase-Glo-3/7, -9 or -8 assays. (**E**) HaCaT cells were pre-treated with DMSO or with 40 µM of the pan-caspase inhibitor Z-VAD-FMK for 30 min. Cells were then infected at a MOI of 5 PFU/cell. After 24 h incubation with DMSO or with Z-VAD-FMK, cell viability was determined by PI staining. Values are the mean of three replicates with error bars indicating standard deviation and *** indicating *p*-values of < 0.001.

To examine LACV-induced cytopathic effects, HaCaT cells were infected at a MOI of 5 PFU/cell and visualized by microscopy. As shown in Figure 2A, cell rounding and detachment from the culture dish were evident as early as 8 hpi, concurrent with the kinetics of LACV N protein expression (Figure 1B). To quantitate cell death, HaCaT cells infected at a high MOI were stained with propidium iodide (PI) at different times post-infection and analyzed by flow cytometry. As shown in Figure 2B, there was no significant PI staining at 8 hpi, however, by 16 hpi, ~30% of cells were PI-positive and this increased to ~40% by 20 hpi. A Cytotox-Glo assay (which measures release of a dead-cell protease) showed similar cytotoxicity at 24 hpi as seen with PI staining and by 36 hpi that toxicity had increased to 70% (Figure 2C). These results indicate that high MOI LACV infection induces significant cell death in HaCaT cells which correlate with the appearance of viral protein in the cells. 

To determine if caspases are activated in HaCaT cells following LACV infection, cells were infected at a high MOI and caspase activity was measured in vitro by Caspase-Glo assays. As shown in Figure 2D, the activity of effector caspases 3/7 were upregulated at 24 hpi to ~10-fold over mock- infected control cells. Likewise, activities of both initiator caspase-8 and caspase-9, were upregulated to ~8-fold at 24 hpi by LACV infection as compared to mock-infected cells. To directly test the role of caspases in LACV-induced cell death, HaCaT cells were pre-treated with the pan-caspase inhibitor Z-VAD-FMK prior to infection at high MOI. At 24 hpi, cell viability was quantitated by PI staining and flow cytometry analysis. Infected cells treated with Z-VAD-FMK had significantly less PI staining, only ~20% of the cells stained positive as compared to 70% in the DMSO control (Figure 2E). Together, these data support the conclusion that LACV induces significant levels of death in HaCaT cells, and death is predominantly mediated by caspase-dependent pathways. 

### 3.3. Multi-Cycle Spread of LACV Infection Is Restricted in HaCaT Cells

**Figure 3:**
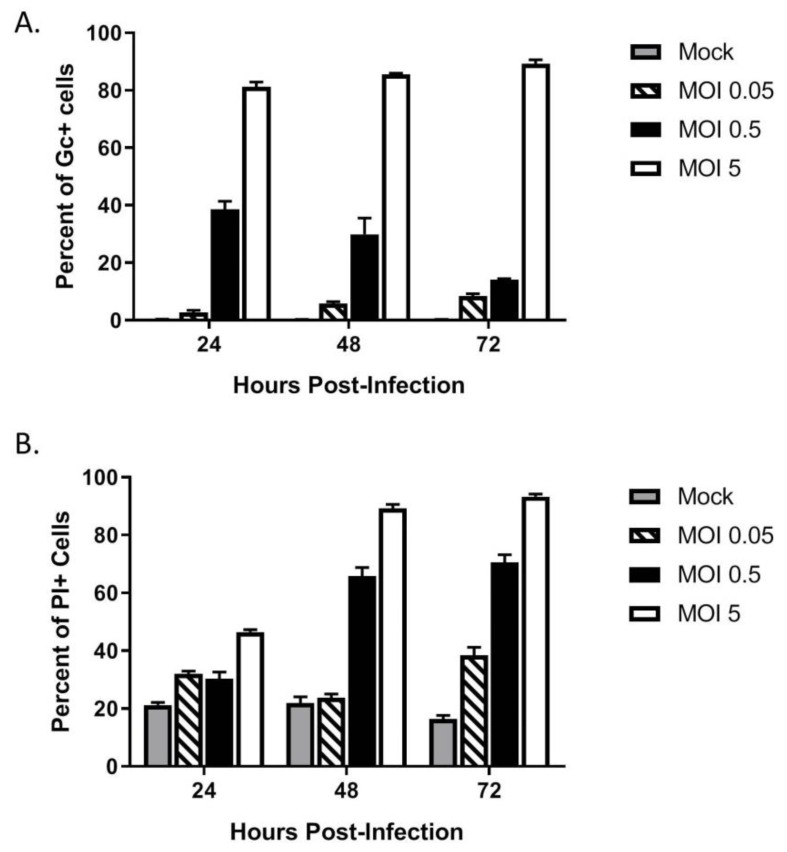
Restricted spread of LACV infection through a population of keratinocytes. HaCaT cells were mock-infected or infected at a MOI of 0.05, 0.5 or 5 PFU/cell. (**A**) At the indicated hpi, cells were analyzed by flow cytometry for LACV Gc expression. Results are expressed as percentage of Gc-positive cells. (**B**) In parallel, cell viability was determined by PI staining. Values are the mean of three replicates with error bars indicating standard deviation.

The above data show that HaCaT cells were permissive to LACV infection at a high MOI. To determine if HaCaT cells supported multi-cycle spread of LACV, HaCat cells were infected at three different MOIs—a high MOI of 5 PFU/cell as baseline for infection, an intermediate MOI of 0.5 PFU/cell, and a low MOI of 0.05 PFU/cell. At indicated timepoints, cells were analyzed by flow cytometry for expression of the LACV Gc glycoprotein. As shown in Figure 3A, low MOI infection with LACV (0.05, striped bars) resulted in very little increase in percentage and number of Gc-positive cells over time, indicating that multi-cycle spread of LACV was restricted. Interestingly, HaCaT cells infected at the intermediate MOI of 0.5 (black bars) showed a distinct profile in the percentage and number of Gc-positive cells, with an initial 40% of the population being Gc-positive at 24 hpi, but a time-dependent decline in this value to ~10% by 72 hpi.

In parallel, LACV-infected HaCaT death over time at different MOIs was assayed using PI staining and flow cytometry. As shown in Figure 3B, mock-infected HaCaT cells had a consistent low-level PI staining (grey bars), whereas high MOI resulted in time-dependent increases in PI staining (white bars). The most striking result was seen with HaCaT cells infected at an intermediate MOI of 0.5 (black bars). While the percentage of Gc-positive cells decreased from 40% at 24 hpi to 10% at 72 hpi, there was a time-dependent increase in PI staining from 30% at 24 hpi to 70% hpi. These data indicate that at a MOI of 0.5 PFU/cell, there is a substantially greater increase in cell death as compared to the number of infected cells.

Together, the above data indicate two consequences of LACV infection of HaCaT cells: (1) LACV is restricted for multi-cycle spread within the HaCaT population, suggesting an antiviral response, and (2) there is increased cell death in the population as compared to the number of cells expressing Gc, suggestive of “bystander” cell death of non-infected cells. In the following sections, we individually address these two components of the HaCaT cell response to LACV infection.

### 3.4. Restriction in Spread through a Population of HaCaT Cells is Due to an Antiviral Response Primarily Driven by Type I IFN

**Figure 4:**
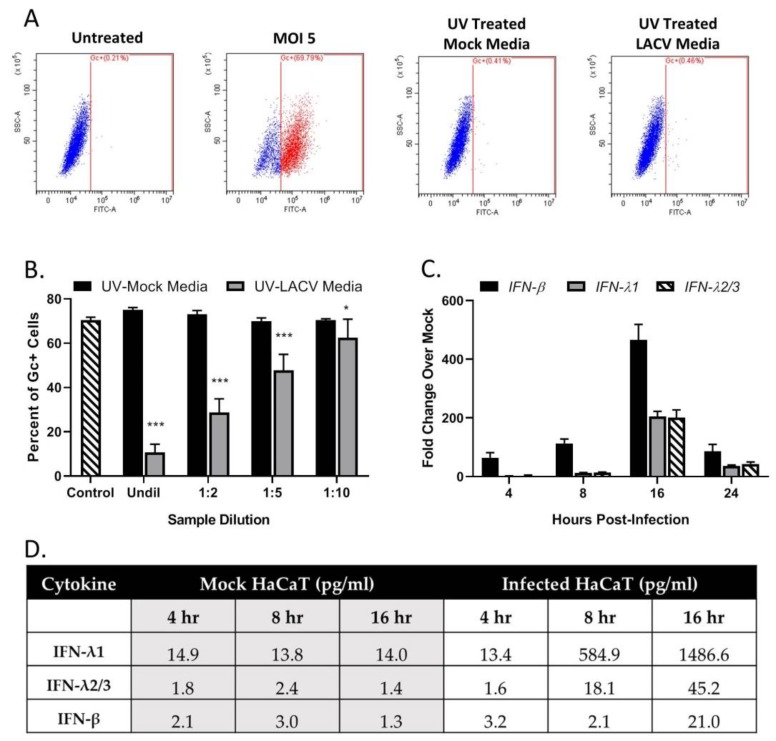
LACV-infected human keratinocytes express antiviral products. HaCaT cells were mock- infected or infected with LACV at a MOI of 5 and media was collected at 8 hpi. (**A**) Media was treated with UV light to inactivate virus and then tested for loss of infectivity on naïve HaCaT cells. At 24 h post treatment, the percentage of infected cells was determined by Gc staining. Untreated and MOI 5 represent negative and positive controls for antibody staining. (**B**) HaCaT cells were treated for 16 h with the indicated dilutions of media from mock-infected (black bars) or LACV-infected cells (gray bars). Cells were then infected with LACV at a MOI of 5 and Gc staining was determined at 8 hpi. Untreated cells were also infected as a control (striped bar). Values are the mean of three replicates with error bars indicating standard deviation and * indicating *p*-values of <0.05 and *** indicating *p*-values of < 0.001. (**C**) HaCaT Cells were infected at a MOI of 5, and total cellular RNA was extracted at the indicated hpi and evaluated for IFN-β, IFN-λ and IFN-λ 2/3 expression by RT-qPCR. (**D**) HaCaT cells were mock-infected or infected with LACV at a MOI of 5 PFU/cell at indicated hpi. Collected Media was treated with UV light to inactivate virus, and cytokine levels were determined by BioLegend’s LEGENDplex™ immunoassay. Values are the average of two samples analyzed in parallel.

A “media transfer” experiment was used to determine if infected cells make products that restrict LACV infection of naïve cells. HaCaT cells were mock-infected or LACV-infected at a MOI of 5 PFU/cell. At 8 hpi, extracellular media was collected and exposed to UV light to inactivate virus. Successful inactivation of virus was evident by the lack of detection of LACV Gc glycoprotein when naïve cells were treated with UV-treated media (Figure 4A). In a separate experiment, naïve HaCaT cells were treated with the UV-inactivated media for 16 h before challenging with LACV at a MOI of 5 PFU/cell. The cells were then analyzed for Gc expression at 8 hpi. As shown in Figure 4B, cells treated with media derived from LACV-infected HaCaT (gray bars) showed a dilution-dependent reduction in Gc expression compared to cells treated with media from mock-infected cells (black bars). To identify potential cytokines produced by LACV-infected HaCaT cells, media was collected from mock-infected or LACV-infected cells at different hpi, treated with UV light, and analyzed by a Biolegend LegendPlex assay. As shown in Figure 4D, mock-infected cells produced basal levels of IFN-λ (both 1 and 2/3) and IFN-β, that did not change substantially over time. By contrast, media from LACV-infected cells contained increasing levels of all three cytokines, and levels peaked at 16 hpi. To determine if LACV infection induced IFN-λ1, IFN-λ2/3 and/or IFN-β at the mRNA level, total RNA was harvested from mock-infected or LACV-infected HaCaT cells and analyzed by qPCR. As shown in Figure 4C, mRNA expression peaked at 16 hpi for all three cytokines, with higher levels of mRNA for IFN-β as compared to IFN-λ.

**Figure 5:**
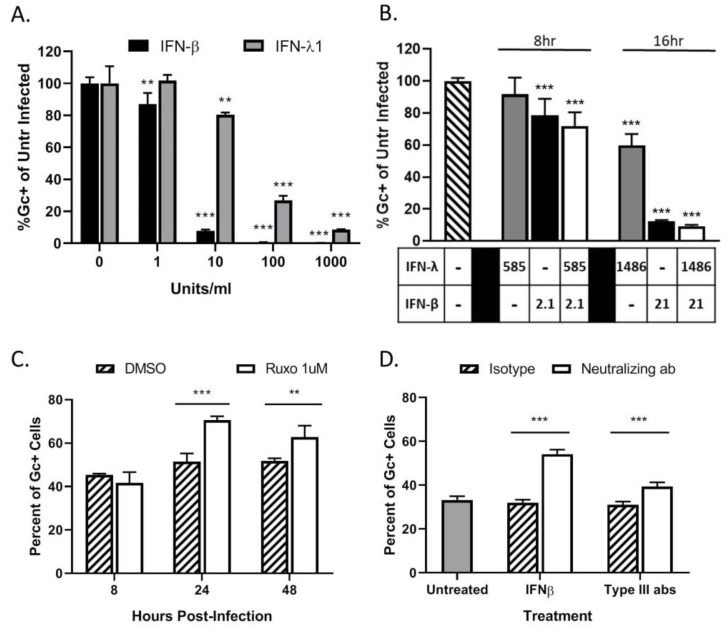
LACV spread through a population of HaCaT cells is limited by type I and type III IFN responses. (**A**,**B**) HaCaT cells were mock-treated or treated for 16 h with the indicated concentrations of exogenous IFN-β or IFN-λ1 (**A**), or a combination of both cytokines (**B**) before infection with LACV at a MOI of 5. At 8 hpi, cells were analyzed by flow cytometry for LACV Gc expression. Data is shown as percentage of untreated infected control. (**C**) HaCaT cells were treated with DMSO as a control or 1 μM Ruxolitinib for 16 h, followed by LACV infection at a MOI of 0.5. At indicated times, cells were analyzed by flow cytometry for Gc expression. (**D**) HaCat cells were first infected at a MOI of 0.5 PFU/cell and were then treated with 3 μg of neutralizing antibodies against IFN-β, a combination of antibodies to Type III IFNs (IL29, IL28a, and Human IFN Lambda Receptor 1) or the corresponding isotype control antibodies. At 24 hpi, cells were analyzed by flow cytometry for LACV Gc expression. In panel D, gray bar represents the percentage of Gc-positive cells in untreated LACV-infected cells. Values are the mean of three replicates with error bars indicating standard deviation and ** indicating *p*-values of <0.01 and *** indicating *p*-values of < 0.001.

To determine if HaCaT cells can respond to IFNs and enter an antiviral state, naïve cells were treated with various concentrations of exogenous IFNs. Cells were then infected at a MOI of 5 PFU/cell with LACV and analyzed by flow cytometry for Gc expression. As seen in Figure 5A, pre-treatment with either type of IFN leads to a significant dose-dependent decrease in percent of Gc-expressing cells as compared to untreated cells. Infection of HaCaT cells was reduced to ~10% by 10 U/mL of IFN-β. In contrast, to reduce infection to the same level, a 100-fold higher concentration (1000 U/mL) of IFN-λ1 was required. 

To determine if concentrations of IFNs released from infected HaCaT cells were sufficient to inhibit LACV infection, cells were treated with IFN levels detected by the LegendPlex assay shown in Figure 4D and then challenged by LACV infection. IFN-β generated at an early time of 8 hpi (2.1 pg/mL) was sufficient to significantly inhibit LACV infection of HaCaT cells, while levels detected at 16 hpi (21 pg/mL) reduced infection to <10%. By contrast, higher levels of IFN-λ1 generated at late times of 16 hpi (1486 pg/mL) were only partially able to inhibit LACV infection (Figure 5B). Importantly, reduction in infection by combined treatment of HaCaT with both cytokines was not significantly different from IFN-β treatment alone, indicating there is little synergistic effect with both IFN-λ1 and IFN-β (Figure 5B).

Type I and Type III IFNs signal through the JAK/STAT pathway to induce an antiviral state. To directly test the role of IFNs in restricting LACV spread, HaCaT cells were treated with 1 μM of the JAK-1/2 inhibitor Ruxolitinib and subsequently infected at a MOI of 0.5 PFU/cell. This MOI was chosen to ensure a significant number of infected (~30–50%) and non-infected cells at early timepoints post-infection. At timepoints shown in Figure 5C, cells were analyzed for Gc expression. At 24 hpi, there was a significant increase in the percentage of Gc-expressing cells after Ruxolitinib treatment (white bars) as compared to control treated cells (cross hatched bars). Since Ruxolitinib can inhibit both type I and type III IFN signaling, neutralizing antibodies were used to distinguish the roles of IFN-β and IFN-λ. HaCaT cells were infected at a MOI of 0.5 PFU/cell and were treated with neutralizing antibodies against IFN-β or against IFN-λ1 and IFN-λ2/3. At 24 hpi, cells were analyzed by flow cytometry for Gc expression. As seen in Figure 5D, there is a significant increase in the percentage of Gc-expression in cells treated with anti-IFN-β (white bar) which had ~50% expression as compared to ~30% in the isotype control (hatched bar). Cells treated with the IFN-λ antibodies also had a small but significant increase, from ~40% to 30%, in the percentage of infected cells as compared to the isotype control.

Taken together, these data show that HaCaT cells produce type I IFN-β and type III IFN-λ in response to LACV infection, which limits the spread of a LACV infection in a population of HaCaT cells. The antiviral response is primarily driven through IFN-β, with a minor contribution by IFN-λ. 

### 3.5. LACV Infection Induces Cell Death in Non-Infected Bystander HaCaT Cells

**Figure 6:**
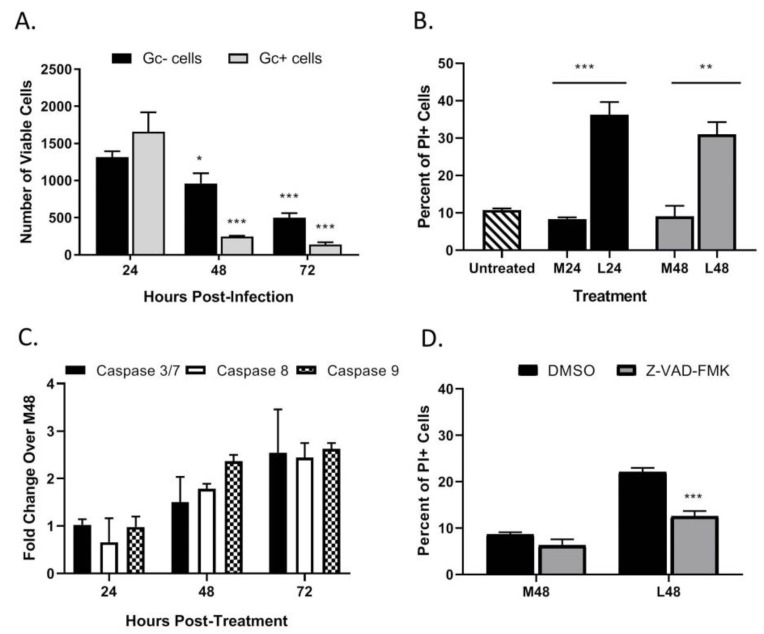
LACV infection of HaCaT cells induces cell death in non-infected bystander cells. (**A**) HaCaT cells were infected at a MOI 0.5 PFU/cell. At the indicated hpi, cells were harvested and analyzed by flow cytometry for LACV Gc expression and for cell viability using Zombie Red™ dye. Data are expressed as number of Gc-positive and Gc-negative viable cells. (**B**–**D**) Media from LACV-infected or mock-infected HaCaT cells were collected at 24 hpi (**B**) and 48 hpi **(B**–**D**) and were UV-treated to inactivate virus. (**B**) Naïve cells were treated for 48 h with UV-inactivated media and cell viability was determined by PI staining. (**C**) Alternatively, cells were treated with UV-inactivated media, and caspase activity was determined by Caspase-Glo-3/7, -9 or -8 assays. Data are expressed as fold change over that seen with cultures treated with media from mock-infected cells (M48). (**D**) Naïve HaCaT cells were pre-treated with 40 μM of Z-VAD-FMK followed by treatment with the indicated media from mock-infected (M48) or LACV-infected cells (L48). After treatment for 48 h, cell viability was determined by PI staining. Values are the mean of three replicates with error bars indicating standard deviation and * indicating *p*-values of <0.05, ** indicating *p*-values of <0.01 and *** indicating *p*-values of < 0.001.

As seen in the above data in Figure 3C, HaCaT cells infected at an intermediate MOI (0.5 PFU/cell) show that ~70% of the population stained positive for PI at 48 hpi, despite only showing ~40% cells being Gc-positive. These data raise the hypothesis that non-infected HaCaT cells in the population are undergoing cell death. To quantitate death of infected vs. non-infected cells, HaCaT cells were infected with LACV at a MOI of 0.5 PFU/cell and at different times post-infection; cells were simultaneously stained with a fixable cell viability dye (Zombie Red™) as well as anti-Gc antibody to quantitate virus-infected cells. In the analysis, Zombie Red was used to exclude non-viable cells and from this, the percentage of Gc was quantitated within the viable population. As shown in Figure 6A, at 24 hpi there were approximately ~1500 viable cells that were Gc-positive and Gc-negative. However, by 72 hpi the number of viable cells was significantly reduced to ~250 Gc-positive cells and ~500 Gc-negative cells. This supports the hypothesis that during LACV infection of HaCaT cells, death occurs in both infected and non-infected cells. 

A media-transfer experiment was used to test the role of extracellular factors in the observed bystander killing during LACV infection. HaCaT cells were mock-infected or infected with LACV at a high MOI and media was collected at 24 (denoted M24 and L24, respectively) and 48 hpi (M48 and L48). Media was exposed to UV light, and virus inactivation was confirmed as described above. Media was then used to treat naïve non-infected HaCaT cells for 48 h and cell viability was quantified by PI staining. As seen in Figure 6B, there is significant cell death in naïve cells treated with media from infected HaCaT cells (L24 and L48) versus those treated with media from mock-infected HaCaT (M24 and M48). These data indicate that LACV-infected HaCaT cells produce factors that can induce cell death in naïve non-infected cells. 

To determine if caspases are activated during bystander cell death, HaCaT cells were treated with UV-inactivated media collected 48 hpi from LACV-infected HaCaT cells, and caspase activity was measured by Caspase-Glo assays. Increased caspase activation was not seen until 48 h post treatment of cells, and by 72 h post treatment there was a ~2–3-fold activation of both iniator caspase -8 and -9, and executioner caspases-3/7 as compared to cells treated with media from mock-infected cells (M48) (Figure 6C). To confirm that observed bystander cell death was caspase-dependent, HaCaT cells were pre-treated with the pan-caspase inhibitor Z-VAD-FMK followed by treatment with UV-inactivated media, and cell viability was quantitated by PI staining after 48 h (Figure 6D). While cell death was significantly reduced by Z-VAD-FMK, PI staining was not reduced to baseline staining induced by control media. These results show that bystander cell death can be induced by factors from LACV-infected HaCaT cells, and this death is due in part to caspase activation.

### 3.6. Bystander Cell Death Observed in Non-Infected HaCaT Cells is IFN-Dependent

**Figure 7:**
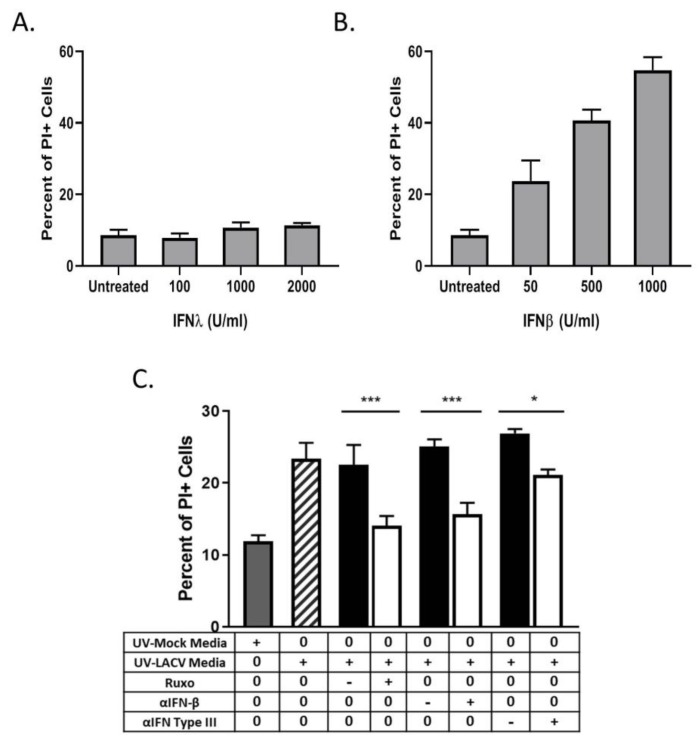
LACV infection induces bystander HaCaT cell death mediated by type I and type III IFN. (**A** and **B**) Naïve HaCaT cells were treated with indicated concentrations of IFN-λ (**A**) or IFN-β (**B**), and cell viability was determined 48 h later by PI staining. **(C)** Media collected at 48 hpi from LACV- infected or mock-infected HaCaT cells was UV-treated to inactivate virus. Naïve cells were pretreated with 1 μM Ruxolitinib or with 3 μg of neutralizing antibodies against IFN-β, or a combination of antibodies that block IL29, IL28a, and Human IFN Lambda Receptor 1 (white bars). Corresponding isotype controls or DMSO controls were also included (black bars). Symbols on the table denote + as treatment, − as corresponding control, and 0 as no treatment. After 48 h of treatment with M48 or L48 media, cell viability was determined by PI staining. Controls included cells treated with UV-inactivated media from mock-infected cells (gray bar) or from LACV-infected cells (striped bar). Values are the mean of three replicates with error bars indicating standard deviation and * indicating *p*-values of <0.05, and *** indicating *p*-values of < 0.001.

We hypothesized that IFNs were responsible for the bystander cell death observed during LACV infection of HaCaT cells. To test this, naïve HaCaT cells were treated with different concentrations of exogenous IFN-λ1 or IFN-β for 48 h and then analyzed for PI staining. At all concentrations tested, IFN-λ1 did not induce any detectable increase in death (Figure 7A). By contrast, IFN-β induced dose-dependent increases in cell death, with 1000 U/mL treatment resulting ~45% PI staining in the population (Figure 7B). To confirm that IFN contributed to the bystander effect, naïve HaCaT cells were treated with UV-inactivated media in the presence of either the Jak 1/2 inhibitor Ruxolitinib or with neutralizing antibodies against IFN-β or all type III IFNs. At 48 h post treatment, cells were analyzed for PI staining. Cells that were treated with UV-inactivated media along with Ruxolitinib or along with IFN neutralizing antibodies had a significant decrease in PI staining as compared to treatment with the corresponding vehicle control or isotype control antibodies (Figure 7C). Together, these data indicate that HaCaT cells are susceptible to IFN-β-induced death, and that IFN-β contributes to bystander death of non-infected cells during multi-cycle LACV infection.

## 4. Discussion

In this study, we have examined the initial replication and spread of LACV through human keratinocytes in culture. Our work was initiated by the need to understand the importance of virus-host cell interactions at the initial site of delivery of insect-borne viruses and the potential for these cells to expand or limit virus replication. HaCaT keratinocytes are highly susceptible to LACV infection at a high MOI, producing high levels of viral proteins and infectious particles as well as inducing extensive cell killing. Unexpectedly, however, multi-cycle replication following low MOI LACV infection is restricted in keratinocyte cultures. Keratinocytes limit spread of LACV infection by mounting a culture-wide antiviral response through the production of both type I and type III IFN. Our most striking finding was that during multi-cycle LACV replication, neighboring non-infected keratinocytes were also killed through IFN-β-dependent and caspase-dependent mechanisms. Based on these findings, we propose a model whereby keratinocytes can serve as a key initial cell type which can limit LACV spread through both induction of an antiviral state as well as killing potential new target cells which could support further centers of replication. 

The HaCaT keratinocyte cells used in this study are from a spontaneously transformed cell line that has been used extensively to study various skin cell processes as well as skin inflammatory conditions, including psoriasis, UV damage and atopic dermatitis [40,41,42,43]. HaCaT cells show normal differentiation patterns and can be induced to secrete various inflammatory cytokines in the same manner as primary keratinocytes [44,45]. In this study, HaCaT cells were maintained at confluencies less than 80% and low passage number for consistency of the culture and to prevent differentiation. Interestingly, we have noted that higher passage cells show phenotypes consistent with differentiation, and these cells show a lower susceptibility to infection with LACV and in their rate and extent of virus-induced death. The role of differentiation of skin cells and arbovirus infection will be an interesting area for future studies.

IFNs have been extensively studied in the context of viral infections as factors that limit virus spread by paracrine and autocrine signaling through a population of cells [46,47,48,49]. In the current study, we show that LACV infection of HaCaT cells results in production of both IFN-β and IFN-λ as early as 8 h after infection, and this correlates with detection of high levels of viral N and Gc proteins. Prior work has shown that the LACV nonstructural protein NSs can prevent type I IFN induction in some cell types by degradation of the RBP1 subunit of RNA polymerase II [50]. Conversely, however, others have reported production of IFN-β in the presence of LACV NSs in cell types such as myeloid dendritic cells and microglial cells [11,51]. Thus, the ability of LACV to prevent induction of antiviral cytokines is very likely a function of differing cell types (e.g., epithelial vs. neuronal vs. keratinocyte) and/or cell species (mouse vs. human) used in a particular study. It is also noteworthy that our time course experiments show that IFN-λ was secreted by LACV-infected HaCaT cells at much higher levels than IFN-β, despite the presence of lower levels of IFN-λ mRNA compared to IFN-β mRNA. These data suggest that the pathways for production of extracellular type I and III IFNs in infected keratinocytes may have major differences, including their regulation at the level of translation or transport, as well as sensitivity to shutoff by viral antagonists (e.g., NSs protein) and cytopathic effects. 

Despite HaCaT cells producing lower levels of IFN-β compared to IFN-λ (e.g., Figure 4D), our antibody neutralization data show that restricted LACV replication was relieved by culturing with the JAK-1/2 inhibitor Ruxolitinib or with a neutralizing antibody to IFN-β. By contrast, IFN-λ pathways did not appear to play a major role in restriction of multi-cycle LACV replication. Similarly, in the absence of virus, naïve HaCaT cells entered an antiviral state capable of blocking LACV infection with much lower levels of IFN-β compared to IFN-λ. It is reported that IFN-β generates a more inflammatory and potent response than IFN-λ [52], which could be due to differences in receptor-ligand affinity, number of IFN receptors, or subsequent downstream signaling events. It is also possible that IFN-λ activates a different landscape of ISGs which are less effective in inhibiting LACV replication compared to those induced by IFN-β [52,53]. Interestingly, inhibition of these two IFN pathways (through Ruxolitinib and with combined neutralizing antibodies) was not sufficient to yield complete unrestricted virus replication throughout the entire population of keratinocyte cells, suggesting that alternative mechanisms besides IFN are in place to restrict multi-cycle LACV replication. 

High MOI LACV infection was very cytopathic to human keratinocyte cells, as evidenced by the visual cell rounding, cytotoxic release of cytoplasmic materials and loss of intact cell membranes (Figure 2A–D). Our data on timing of cell killing and IFN induction after high MOI LACV infection are consistent with previous reports of LACV-induced killing of mouse neuronal cells through common RIG-I and MAVS pathways that activate both cytokine production as well as apoptosis [54]. In addition, others have shown that LACV NSs bears some sequence and functional similarities to drosophila reaper, a protein that induces apoptosis by translational shutoff and cytochrome C release [55], suggesting a possible role in mimicking a cellular inducer of death. Keratinocyte cell killing by LACV was dependent on functions of caspase pathways, but the death pathways are likely to be complex and overlapping since both caspase-8 and -9 were induced simultaneously.

Our most striking finding emerged from results that multi-cycle LACV infections at low MOIs led to higher amounts of keratinocyte cell death than could be accounted for by the percentage of infected cells. Our dual staining of LACV-infected populations as well as media-transfer experiments showed (1) non-infected cells within the HaCaT population were dying, (2) that this death was similar to LACV-infected cells by being at least in part caspase-dependent, and (3) that death was induced by extracellular factors secreted from the LACV-infected HaCaT cell population. Remarkably, naïve HaCaT cells were killed by treatment with IFN-β alone but not by IFN-λ treatment, and inhibition of IFN signaling with Ruxolitinib and with IFN-β neutralizing antibody reduced killing by media from LACV-infected cells. Our experiments suggest that at early times after infection, IFN-β exerts a protective role in the population which prevents further spread of the infection, but at later times post-infection continuous IFN-β signaling has a pro-apoptotic effect on non-infected neighboring cells. Other work has shown that extended IFN signaling can lead to differential regulation of STAT protein expression and this can play a role in cell survival versus cell death [52]. Several studies have shown that type I IFNs can induce cell death in various types of cells through different pathways. For example, IFN-β treatment led to death of neuroblastoma cells through activation of the OAS/RNAseL system, activation of PKR, and differential regulation of STATs as well as inhibition of pro-survival signaling by the PI3k/AKT pathway [56,57]. IFN-β induced cell death has also been studied in the context of cancer therapies. For example, IFN-β-mediated death of human cervical carcinoma cells can be through changes in the caspase-8/cFLIP balance in the death-inducing signaling complex (DISC) [58]. It has been proposed that IFN may act through activation of NF-κb and subsequent expression of pro-apoptotic proteins such as Fas, Bax, and p53 [57]. It is important to note that under the conditions of our experiments, death of LACV-infected cells may be driven by both extracellular IFN-β autocrine signaling and by intracellular responses to LACV replication. Likewise, we have shown that death of non-infected bystander cells is driven by IFN-β, but it may also be influenced by the presence of other cellular or viral components (e.g., UV-inactivated virions or proteins) that are released from infected cells. Future studies on IFN-β-mediated cell death pathways will elucidate the role that each plays in the bystander cell death effect observed in LACV infections.

We propose a model whereby LACV is introduced by insect bite into the skin tissue, where it can initially infect keratinocytes. As a result, keratinocytes mount an innate immune response that places nearby cells in an antiviral state and also induces cell death in the nearby non-infected cells as a mechanism to prevent further spread of LACV to new cells. Despite this antiviral response, the rapid LACV replication cycle (less than 12 h) allows production of new virions before antiviral mechanisms are fully in place. Newly released virions can then disseminate to other tissues such as the muscle, plasma, and eventually the nervous system. Further studies are needed to understand the role of other local skin cells, such as dermal fibroblasts and resident immune cells, in the initial infection of LACV. Elucidating these initial interactions with host skin cells in vitro could contribute to a better understanding of determinants of host susceptibility to LACV infection and disease. 

## Figures and Tables

**Table 1:** Primers.

	Forward Primer	Reverse Primer
**β-actin**	5′-GATCATTGCTCCTCCTGAGC-3′,	5’-ACTCCTGCTTGCTGATCCAC-3,
**IFN-β**	5’-CAGCTCTTTCCATGAGCTACAA-3’	5’-CAGTATTCAAGCCTCCCATTCA-3’
**IFN-λ1**	5’-CTTGGACCGTGGTGCTG-3’	5’-CAGCCCTTCCCAGTTGTG-3’
**IFN-λ2/3**	5’-CAGTGCTGGTGCTGATG-3’	5’-GACTTGAACTGGGCTATGTG-3’
